# Healing of joint capsule after hip arthroscopy using interportal capsulotomy and capsular closure influences clinical outcomes

**DOI:** 10.1186/s13018-022-03208-z

**Published:** 2022-06-15

**Authors:** Guanying Gao, Chenbo Jiao, Jiayang Liu, Chang Zhou, Yuhao Liu, Yingfang Ao, Yan Xu

**Affiliations:** Institute of Sports Medicine, Peking University Third Hospital, 49 North Garden Road, Haidian District, Beijing, 100191 China

**Keywords:** Hip, Femoroacetabular impingement, Capsule, Magnetic resonance imaging

## Abstract

**Background:**

Hip arthroscopy for treatment of femoroacetabular impingement (FAI) has developed rapidly and has been shown to significantly decrease pain and improve hip function. However, the relationship between hip capsule characteristics and healing after arthroscopic surgery and changes in patient-reported outcomes scores (PROs) for postoperative pain, function, and symptoms is still uncertain.

**Methods:**

We retrospectively evaluated consecutive patients who were diagnosed with FAI and underwent hip arthroscopy for treatment in our hospital between May 2018 and November 2020. All patients had preoperative MRI and postoperative MRI at least 6 months after arthroscopy. Hip capsular thickness was measured at the proximal, middle, and distal site of the capsule. PROs and PROs at final follow-up were obtained, including visual analog scale (VAS) for pain and modified Harris Hip Score (mHHS).

**Results:**

A total of 194 patients were included in this study. The mean MRI follow-up time was 14.3 (range, 6–37) months, and the mean clinical follow-up time was 26.1 (range, 12–43) months. Postoperative capsular thickness or net change were not correlated with postoperative PROs and VAS (*P *> .05). Capsular defect was observed in 17 (8.8%) patients. Patients with capsular defect had a relatively higher BMI (*P* < .05). Patients with capsular defect had a significant lower mHHS and higher VAS compared with patients with continuous capsule (*P* < .05). Ninety-one percentage of patients with continuous capsule surpassed minimal clinically important difference (MCID) and 80.8% achieved PASS, but only 58.8% of patients with capsular defect surpassed MCID and 47.1% achieved patient acceptable symptom state (PASS).

**Conclusions:**

Postoperative capsular thickness may not have influence on the clinical outcomes of hip arthroscopy for treatment of FAI. Some capsule of patients who underwent arthroscopic interportal capsulotomy and repair could not heal. Postoperative capsular continuity had a great impact on the clinical outcomes of hip arthroscopy for FAI. Patients with higher BMI may be more likely to have capsule failure to heal.

## Introduction

Over the past two decades, hip arthroscopy has developed rapidly and has been shown to significantly decrease pain and improve hip function [1–6]. However, there is a lack of consensus regarding proper capsular management during hip arthroscopy and the influence of capsule healing and characteristics on the clinical outcomes of hip arthroscopy is still unknown [7–10]. The iliofemoral ligament is the strongest of the ligaments comprising the hip capsule and plays a significant role in hip stability [11, 12]. Shaw et al. found that hip capsule morphology correlates with patient symptoms in the setting of femoroacetabular impingement (FAI) as increased anterior capsular volume is associated with greater patient pain [13]. Nguyen et al. thought changes in hip capsule morphology including decreased anterior–posterior capsule thickness ratio after surgery may be correlated with improvements in patient pain, function, and ability to return to sports [7]. Hip capsule was also reported to be related to iatrogenic instability following hip arthroscopy [14, 15]. However, the sample size of the current research is too small to explain how hip capsule characteristics and healing after arthroscopic surgery are correlated with changes in patient-reported outcomes scores (PROs) for postoperative pain, function, and symptoms.

The purpose of this study was to evaluate the influence of characteristics and healing of joint capsule after hip arthroscopy for FAI on the clinical outcomes through magnetic resonance imaging (MRI) and clinical follow-up. We hypothesized that postoperative capsular thickness and continuity could have influence on PROs.

## Methods

### Patients

We evaluated consecutive patients who were diagnosed with FAI and underwent hip arthroscopy for treatment in our hospital between May 2018 and November 2020. The inclusion criteria were as follows: (1) patients who were diagnosed with FAI by clinical findings, plain radiographs, computed tomography (CT), and MRI; and (2) underwent hip arthroscopy for treatment; and (3) had preoperative MRI and postoperative MRI at least 6 months after arthroscopy. Patients with prior hip surgery and patients who could not complete the MRI and clinical follow-up were excluded from the study. All participants signed informed consent. The study was approved by the Ethics Committee of the Third Hospital of Peking University. All methods were performed in accordance with the guidelines and regulations of the Ethics Committee of the Third Hospital of Peking University.

### Surgical technique

All surgeries were performed using a standard supine approach as described by Gao et al. [16]. In brief, the interportal capsulotomy technique was used to access the hip joint using the anterolateral and midanterior portals. A detailed inspection of the central compartment was performed to assess the acetabular rim, acetabular labrum, articular cartilage, and ligamentum teres. Labral repair or labral debridement was performed according to the nature of injury. Femoral osteoplasty or acetabuloplasty was performed according to the intraoperative findings. Focal subspinal decompression or partial resection of the lesser trochanter were performed when combined subspine impingement (SSI) or ischiofemoral impingement (IFI) was identified. Capsular closure was routinely done at the end of surgery. Capsular closure was performed with 2 or 3 #2 Orthocord sutures (DePuy Mitek, Raynham, MA).

### MRI evaluation of hip capsule

**Fig. 1:**
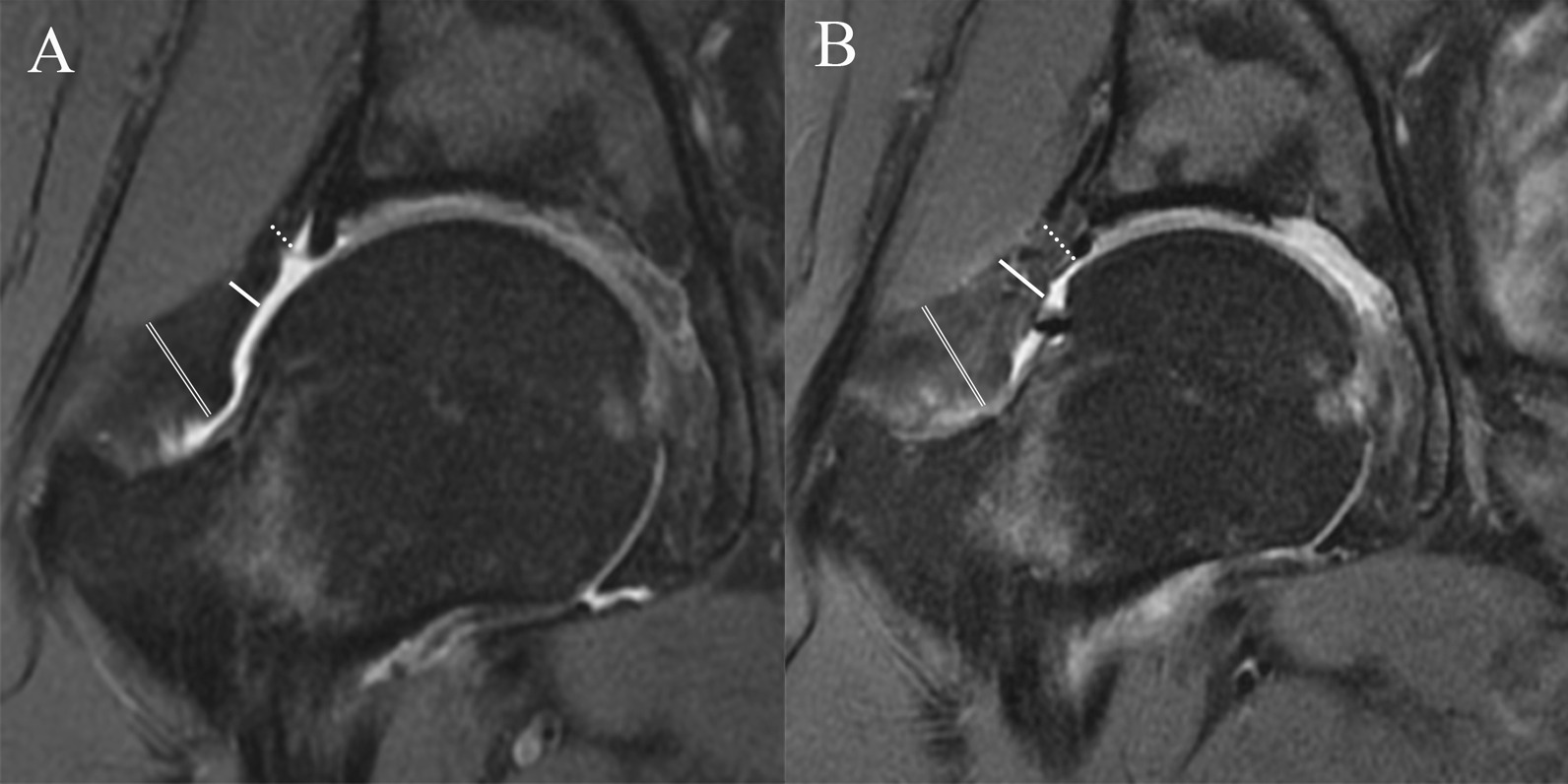
**A** Preoperative measurements of capsular thickness at the proximal (dotted line), middle (single solid line), and distal (double line) site. **B** Measurements of capsular thickness at the proximal (dotted line), middle (single solid line), and distal (double line) site in the same patient in MRI follow-up

Two musculoskeletal fellowship-trained doctors performed all hip capsule measurements. The doctors were blinded to the clinical and operative findings and to the each other’s findings to prevent potential bias. The hip MRI was performed with a 3.0 T MR scanner (Magnetom Trio with TIM system, Siemens Healthcare) and a dedicated flexible surface coil around the affected hip joint as described by Gao et al. [17]. As described by Strickland et al. [14], hip capsular thickness was measured in the midcoronal plane to the femoral head on the coronal fat-saturated proton density (FSPD) sequence at 3 sites: at the level of the femoral head–neck junction (midcapsular thickness), at a point midway between the midpart of the capsule and the labrum (proximal capsular thickness), and at a point equidistant toward the greater trochanter (distal capsular thickness) (Fig. 1). Capsular thickness was calculated by measuring the low-signal-intensity substance of the capsule between the articular side and the muscular side. Capsule continuity was also evaluated.

### Radiographic and clinical evaluation

Preoperative alpha angle and lateral center–edge angle (LCEA) were measured as described by previous studies [18, 19]. Preoperative PROs and PROs at final follow-up were obtained, including visual analog scale (VAS) for pain and modified Harris Hip Score (mHHS). Complications or revision hip arthroscopy were recorded. For the mHHS, the minimal clinically important difference (MCID) was defined as 8 by Kemp et al. [20], and the patient acceptable symptom state (PASS) score was defined as 74 by Chahal et al. [21].

### Statistics

The two-tailed paired t test was used to evaluate significance between preoperative and postoperative PROs and capsule thickness. Percentages were compared using the Chi-square test. Interrater reliability was evaluated using a two-way, mixed, absolute-agreement, single-measures intraclass correlation coefficient (ICC). Multivariate logistic regression models were built to determine the effect of independent variables (age, sex, BMI, capsular defect, preoperative and postoperative capsular thickness) on achieving MCID thresholds. *P* values < 0.05 were considered statistically significant. All statistical analyses were performed with SPSS Statistics, version 22 (IBM).

## Results

As shown in Table 1, a total of 194 patients (mean age, 37.1 years; age range, 15–65 years; 88 males and 106 females) were included in this study. Sides, body mass index (BMI), alpha angle, LCEA, MRI follow-up time, and clinical follow-up time are given in Table 1. Arthroscopic procedures are given in Table 2. There were no complications or revision hip arthroscopy reported in this study.

**Table 1:** Demography of patients (*n* = 194)

Parameter	Data
Age, y, mean (range)	37.1 (15–65)
*Sex*	
Male	88 (45.4%)
Female	106 (54.6%)
*Side*	
Left	76 (39.2%)
Right	118 (60.8%)
BMI, kg/m^2^, mean (range)	23.1 (16.0–35.7)
Alpha angle, mean ± SD	58.7 ± 6.8
LCEA, mean ± SD	34.2 ± 7.3
MRI follow-up time, month, mean (range)	14.3 (6–37)
Clinical follow-up time, month, mean (range)	26.1 (12–43)
*Preoperative capsular thickness, mm, mean* ± *SD*	
Proximal	3.8 ± 1.4
Middle	4.9 ± 1.7
Distal	7.3 ± 2.0
*Postperative capsular thickness, mm, mean* ± *SD*	
Proximal	4.7 ± 1.6
Middle	6.3 ± 2.0
Distal	8.6 ± 2.0

Unless otherwise specified, data are numbers of patients, with percentages in parentheses

*SD* Standard deviation

**Table 2:** Arthroscopic procedures

Procedures	Number (%)
Femoral osteoplasty	192 (99.0)
Acetabuloplasty	136 (70.1)
Labral debridement	13 (6.7)
Labral repair	178 (91.8)
Labral reconstruction	3 (1.5)
Focal subspinal decompression	35 (18.1)
Lesser trochanter resection	7 (3.6)
Capsular plication	18 (9.3)
Capsular closure	194 (100)

Preoperative and postoperative capsular thickness at the proximal, middle, and distal site of the capsule are given in Table 1. There was significant increase in postoperative capsular thickness compared with preoperative capsular thickness at the proximal, middle, and distal site of the capsule (proximal, *P* = 3.93E−07; middle, *P* = 1.00E−08; distal, *P* = 7.07E−09). The ICC for preoperative capsular thickness at the proximal, middle, and distal site of the capsule between 2 evaluators was 0.75, 0.84, and 0.81, respectively. The ICC for postperative capsular thickness at the proximal, middle, and distal site of the capsule between 2 evaluators was 0.81, 0.86, and 0.84, respectively.

**Fig. 2:**
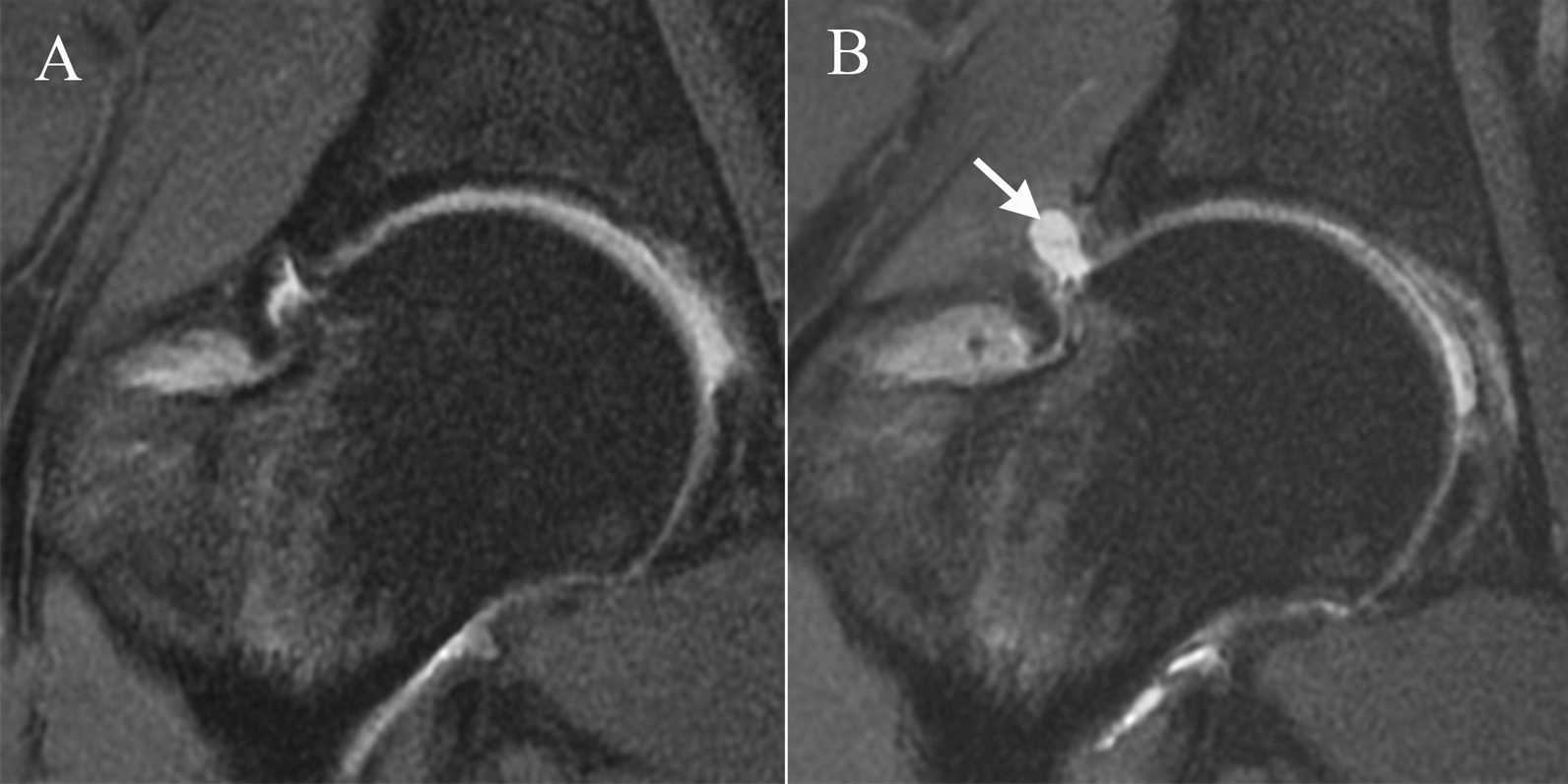
**A** Preoperative hip MRI showed continuous capsule. **B** MRI at 14 months after hip arthroscopy showed capsular defect (white arrow) in the same patient

Capsular defect was observed in 17 (8.8%) patients (Fig. 2). There was no significant difference in sex, age, preoperative capsular thickness, alpha angle, LCEA, and preoperative PROs between patients with capsular defect and patients with continuous capsule. Patients with capsular defect had a relatively higher BMI (24.8 ± 4.7 in patients with capsular defect vs 22.8 ± 3.5 in patients with continuous capsule, *P* = 0.041).

As shown in Table 3, preoperative mHHS and VAS were 48.4 ± 14.3 (range, 18–65) and 4.7 ± 1.9 (range, 1–9), respectively. Postperative mHHS and VAS were 78.2 ± 12.3 (range, 42–91) and 1.2 ± 1.2 (range, 0–6), respectively. Both mHHS and VAS had significant improvement (mHHS, *P* = 4.09E−09; VAS, *P* = 3.21E−07). Postoperative mHHS of patients with capsular defect and patients with continuous capsule was 69.5 ± 14.3 (range, 42–87) and 79.2 ± 13.4 (range, 59–91), respectively. Patients with capsular defect had a significant lower mHHS compared with patients with continuous capsule (*P* = 5.65E−5). Postoperative VAS of patients with capsular defect and patients with continuous capsule was 3.6 ± 1.4 (range, 2–6) and 1.0 ± 1.1 (range, 0–6), respectively. Patients with capsular defect had a significant higher VAS compared with patients with continuous capsule (*P* = 2.13E−3). Postoperative capsular thickness or net change at the proximal, middle, and distal site were not correlated with postoperative PROs and VAS (thickness with mHHS: proximal, *P* = 0.121, middle, *P* = 0.323, distal, *P* = 0.213; thickness with VAS: proximal, *P* = 0.541, middle, *P* = 0.611, distal, *P* = 0.312; net change with mHHS: proximal, *P* = 0.687, middle, *P* = 0.719, distal, *P* = 0.690; net change with VAS: proximal, *P* = 0.416, middle, *P* = 0.520, distal, *P* = 0.492). In all 177 patients with continuous capsule, 161 (91.0%) patients surpassed MCID and 143 (80.8%) patients achieved PASS. In all 17 patients with capsular defect, 10 (58.8%) patients surpassed MCID and 8 (47.1%) patients achieved PASS. After we used multivariate logistic regression models to determine the effect of independent variables on achieving MCID thresholds, we found capsular defect was associated with failure of achieving MCID. These predictions were noted using odds ratios with 95% confidence intervals. The results of these analyses are presented in Table 4.

**Table 3:** Preoperative and postoperative PROs and VAS

	Pre-op mHHS	Post-op mHHS	Pre-op VAS	Post-op VAS
All patients	48.4 ± 14.3	78.2 ± 12.3	4.7 ± 1.9	1.2 ± 1.2
Patients with continuous capsule	49.3 ± 14.6	79.2 ± 13.4	4.4 ± 2.0	1.0 ± 1.1
Patients with capsular defect	48.0 ± 11.3	69.5 ± 14.3	4.8 ± 1.3	3.6 ± 1.7

Data is mean ± standard deviation

**Table 4:** Multivariate logistic regression model to identify significant predictors of achieving MCID

	MCID		
	OR	CI (95%)	*P* value
Age, y	1.00	0.98–1.02	0.89
*Sex*			
Female	Reference		
Male	0.91	0.72–1.22	0.69
BMI, kg/m^2^	0.59	0.34–1.01	0.060
*Capsular defect*			
No defect	Reference		
Defect	0.47	0.28–0.79	0.004
*Preoperative capsular thickness*			
Proximal	1.16	0.57–2.39	0.679
Middle	1.19	0.87–1.64	0.292
Distal	1.09	0.93–1.21	0.302
*Postperative capsular thickness*			
Proximal	1.10	0.63–1.51	0.479
Middle	1.07	0.89–1.54	0.312
Distal	1.01	0.72–1.22	0.611

## Discussion

In this study, we found that capsular thickness increased after arthroscopic interportal capsulotomy and repair compared with preoperative capsular thickness at the proximal, middle, and distal site of the capsule. About 8.8% of the capsule of patients who underwent arthroscopic interportal capsulotomy and repair could not heal. Postoperative capsular continuity had a great impact on the clinical outcomes of hip arthroscopy for FAI. Patients with higher BMI may be more likely to have capsule failure to heal.

At present, the influence of capsule healing and characteristics on the clinical outcomes of hip arthroscopy for treatment of FAI is still unknown. Shaw et al. evaluated 35 patients with symptomatic FAI and concluded that increased anterior capsular volume is associated with greater preoperative pain [13]. However, this study did not mention postoperative PROs. Nguyen et al. evaluated 28 patients who underwent hip arthroscopy through periportal capsulotomy without closure and concluded that decreased anterior–posterior capsule thickness ratio after surgery may be correlated with improvements in patient pain, function, and ability to return to sports [7]. At present, this is the only one study which has mentioned the relationship between capsular characteristics and postoperative PROs. However, the sample size of the current research is too small to explain how hip capsule characteristics and healing after arthroscopic surgery are correlated with postoperative PROs.

In our study, the capsular thickness increased after arthroscopic interportal capsulotomy with capsular repair compared with preoperative capsular thickness at the proximal, middle, and distal site of the capsule. This is consistent with the results of a prior study, which evaluated 39 patients and reported that the hip capsule adjacent to the capsulotomy and subsequent repair is thickened compared with the same location on the contralateral, nonoperative hip [22]. However, Nguyen et al. reported a decrease in anterior and posterior hip capsular thickness [7]. It should be noticed that in both our study and the study conducted by Weber et al., the entire capsulotomy was repaired with high-strength mattress sutures. In the study conducted by Nguyen et al., hip arthroscopy was performed through periportal capsulotomy without closure [7]. This showed that different capsular management during hip arthroscopy may have an influence on the postoperative capsular thickness and no repairing of capsule may result in thinning of the capsule. Combined with the results of our study, we suggest routine hip capsule closure after hip arthroscopy. Further study is needed to study the relationship between capsular management and postoperative capsular thickness.

It should be noticed that capsular defect was observed in 8.8% patients in our study. Weber et al. evaluated 39 patients and observed 3 (7.5%) capsular defects after arthroscopic capsulotomy and capsular repair [22]. This percentage is similar to our study. Nguyen et al. evaluated 28 hips and reported all patients had healed periportal capsulotomies without capsular defects in the coronal or axial–oblique planes on postoperative MRI at 1 year [7]. Strickland et al. evaluated 17 patients who underwent hip arthroscopy with or without capsular repair on postoperative MRI at 24 weeks and reported all patients demonstrated progression to healing, with a contiguous appearance without defects [14]. These two studies reported a 100% capsule healing rate. However, a small sample size of these two studies may not be enough to explain this problem. Capsular healing had a regular failure rate, even if the entire capsulotomy was repaired with high-strength mattress sutures.

In our study, we found that postoperative capsular continuity had a great impact on the clinical outcomes of hip arthroscopy for FAI. Patients with capsular defect had a significant lower mHHS compared with patients with continuous capsule. Ninety-one percentage of patients with continuous capsule surpassed MCID and 80.8% achieved PASS, but only 58.8% of patients with capsular defect surpassed MCID and 47.1% achieved PASS. This showed the importance of the capsular continuity to the clinical outcomes of arthroscopy for FAI. We should pay more attention to capsular healing and the method of hip capsular closure in our daily work.

The factors affecting capsular healing were still unclear. In our study, we found that patients with higher BMI may be more likely to have joint capsule failure to heal. However, there was no significant difference in sex, age, preoperative capsular thickness, alpha angle, LCEA, and preoperative PROs between patients with capsular defect and patients with continuous capsule. Further study is needed to find out the factors affecting capsular healing and increase the healing rate of capsule after hip arthroscopy.

## Limitation

This study has several limitations. Firstly, clinical follow-up and MRI follow-up are not performed at the same time. Clinical follow-up was performed 3–12 months after MRI follow-up in order to eliminate the influence of postoperative rehabilitation process. Previous study has shown that the capsule will heal within 24 weeks [14]. All MRI follow-up was performed more than 6 months after operation in our study, so we thought it had little influence on the evaluation of capsular healing. Secondly, the time points of MRI follow-up are different, and the thickness of capsule may be different at different time points. Further study is needed to find out the change of capsular thickness with time. Thirdly, although the large sample size provided a substantial amount of evidence to support the findings, the retrospective nature of this study constituted inherent potential limitations.

## Conclusion

Postoperative capsular thickness may not have influence on the clinical outcomes of hip arthroscopy for treatment of FAI. Some capsule of patients who underwent arthroscopic interportal capsulotomy and repair could not heal. Postoperative capsular continuity had a great impact on the clinical outcomes of hip arthroscopy for FAI. Patients with higher BMI may be more likely to have capsule failure to heal.

## Data Availability

Not applicable.

## Abbreviations

FAI: Femoroacetabular impingement
MCID: Minimal clinically important difference
PASS: Patient acceptable symptom state
